# Supplementary material for: Ad26.COV2.S protects Syrian hamsters against G614 spike variant SARS-CoV-2 and does not enhance respiratory disease
Source: NPJ Vaccines. 2021 Mar 19;6:39. doi: 10.1038/s41541-021-00301-y (PMC7979827; doi:10.1038/s41541-021-00301-y)
Supplement: Supplementary file 2 — Reporting Summary [file 41541_2021_301_MOESM2_ESM.pdf]

nature research

Corresponding author(s): J.E.M. van der Lubbe
Jvander7@its.jnj.com

Last updated by author(s): Feb 2, 2021

Reporting Summary

Nature Research wishes to improve the reproducibility of the work that we publish. This form provides structure for consistency and transparency in reporting. For further information on Nature Research policies, see our Editorial Policies and the Editorial Policy Checklist.

Statistics

For all statistical analyses, confirm that the following items are present in the figure legend, table legend, main text, or Methods section.

n/a	Confirmed	
☐	☒	The exact sample size (*n*) for each experimental group/condition, given as a discrete number and unit of measurement
☐	☒	A statement on whether measurements were taken from distinct samples or whether the same sample was measured repeatedly
☐	☒	The statistical test(s) used AND whether they are one- or two-sided<br>*Only common tests should be described solely by name; describe more complex techniques in the Methods section.*
☐	☒	A description of all covariates tested
☐	☒	A description of any assumptions or corrections, such as tests of normality and adjustment for multiple comparisons
☐	☒	A full description of the statistical parameters including central tendency (e.g. means) or other basic estimates (e.g. regression coefficient) AND variation (e.g. standard deviation) or associated estimates of uncertainty (e.g. confidence intervals)
☐	☒	For null hypothesis testing, the test statistic (e.g. *F*, *t*, *r*) with confidence intervals, effect sizes, degrees of freedom and *P* value noted<br>*Give P values as exact values whenever suitable.*
☒	☐	For Bayesian analysis, information on the choice of priors and Markov chain Monte Carlo settings
☒	☐	For hierarchical and complex designs, identification of the appropriate level for tests and full reporting of outcomes
☒	☐	Estimates of effect sizes (e.g. Cohen's *d*, Pearson's *r*), indicating how they were calculated

Our web collection on statistics for biologists contains articles on many of the points above.

Software and code

Policy information about availability of computer code

Data collection	n/a
Data analysis	Statistical analyses were performed using SAS version 9.4 (SAS Institute Inc. Cary, NC, US) and R version 3.6.1 (2019-07-05).

For manuscripts utilizing custom algorithms or software that are central to the research but not yet described in published literature, software must be made available to editors and reviewers. We strongly encourage code deposition in a community repository (e.g. GitHub). See the Nature Research guidelines for submitting code & software for further information.

Data

Policy information about availability of data

All manuscripts must include a data availability statement. This statement should provide the following information, where applicable:
- Accession codes, unique identifiers, or web links for publicly available datasets
- A list of figures that have associated raw data
- A description of any restrictions on data availability

All data that support the findings of this study are available from the corresponding author upon reasonable request.

Field-specific reporting

Please select the one below that is the best fit for your research. If you are not sure, read the appropriate sections before making your selection.

☒ Life sciences ☐ Behavioural & social sciences ☐ Ecological, evolutionary & environmental sciences

For a reference copy of the document with all sections, see nature.com/documents/nr-reporting-summary-flat.pdf

Life sciences study design

All studies must disclose on these points even when the disclosure is negative.

Sample size	Each study had a predefined objective, and based on historical data obtained in-house, sample sizes were calculated by statisticians to detect predetermined effect sizes with 80% power.
Data exclusions	No data was excluded during analysis
Replication	All experiments included either control groups or control time-points validating the experiments. All experiments met the pre-set gatekeepers for the validity of the experiment. Experiments were not replicated due to ethical reasons of animal experimentation.
Randomization	Hamsters and rabbits were randomly assigned to groups
Blinding	Investigators were blinded to group allocation during in vivo and in vitro data collection. Blinding was removed during data analysis after the experiment finished and results had been quality controlled

Reporting for specific materials, systems and methods

We require information from authors about some types of materials, experimental systems and methods used in many studies. Here, indicate whether each material, system or method listed is relevant to your study. If you are not sure if a list item applies to your research, read the appropriate section before selecting a response.

Materials & experimental systems

n/a	Involved in the study	
☐	☒	Antibodies
☐	☒	Eukaryotic cell lines
☒	☐	Palaeontology and archaeology
☐	☒	Animals and other organisms
☒	☐	Human research participants
☒	☐	Clinical data
☒	☐	Dual use research of concern

Methods

n/a	Involved in the study	
☒	☐	ChIP-seq
☒	☐	Flow cytometry
☒	☐	MRI-based neuroimaging

Antibodies

Antibodies used	Rabbit-Anti-Hamster IgG HRP (Invitrogen, catalogue number A18895) and anti-rabbit IgG-HRP (Jackson ImmunoResearch, catalogue number 111-035-046)
Validation	the antibodies were authenticated according to the CoA of the manufacturers

Eukaryotic cell lines

Policy information about cell lines

Cell line source(s)	Vero E6 cells; CRL-1580, American Type Culture Collection (ATCC)
Authentication	the cell line was authenticated according to the CoA of the manufacturer
Mycoplasma contamination	All cell lines tested negative for mycoplasma contamination
Commonly misidentified lines (See ICLAC register)	N.A.

Animals and other organisms

Policy information about studies involving animals; ARRIVE guidelines recommended for reporting animal research

Laboratory animals	Male Syrian (golden) hamsters (Mesocricetus auratus), strain HsdHan:AURA, aged 9-11 weeks at the start of the study were purchased from Envigo (Envigo RMS B.V., Venray, the Netherland). Female New Zealand White rabbits, aged approximately 4 months at the start of the study were purchased from Charles River Laboratories in France.
Wild animals	The study did not involve wild animals
Field-collected samples	The study did not involve samples collected from the field
Ethics oversight	Animal experiments were approved by the Central Authority for Scientific Procedures on Animals (Centrale Commissie Dierproeven) and conducted in accordance with the European guidelines (EU directive on animal testing 86/609/EEC) and local Dutch legislation on animal experiments.

Note that full information on the approval of the study protocol must also be provided in the manuscript.
